# Mechanical Characteristics of the Flebogrif System—The New System of Mechano-Chemical Endovenous Ablation

**DOI:** 10.3390/ma15072599

**Published:** 2022-04-01

**Authors:** Piotr Terlecki, Marek Boryga, Paweł Kołodziej, Krzysztof Gołacki, Zbigniew Stropek, Dariusz Janczak, Maciej Antkiewicz, Tomasz Zubilewicz

**Affiliations:** 1 Chair and Department of Vascular Surgery and Angiology, Medical University of Lublin, 20-081 Lublin, Poland; piotrterlecki@interia.pl (P.T.); tomzubil@poczta.onet.pl (T.Z.); 2 Department of Mechanical Engineering and Automation, Faculty of Production Engineering, University of Life Sciences in Lublin, 20-612 Lublin, Poland; pawel.kolodziej@up.lublin.pl (P.K.); krzysztof.golacki@up.lublin.pl (K.G.); 3 Department of Vascular Surgery, General and Transplant Surgery, Wroclaw Medical University, 50-556 Wrocław, Poland; dariusz.janczak@op.pl (D.J.); maciej.antkiewicz@gmail.com (M.A.)

**Keywords:** veins ablation, mechanical characteristics, non-thermal ablation, Flebogrif

## Abstract

Non-thermal endovenous ablations, due to the lowest probability of complications, are the new method of treating chronic venous insufficiency—one of the most common diseases globally. The Flebogrif system (Balton Sp. z o.o., Warsaw, Poland) is a new mechano-chemical ablation system causing the mechanical damage of endothelium that allows for better sclerosant penetration into its wall. The purpose of the article is to provide mechanical characteristics in the form of force–displacement dependence for a single cutting element, and a bundle of cutting elements of Flebogrif as a whole for different levels of protrusion of the bundle of cutting elements. A TA.HD plus (Stable Micro Systems, Godalming, UK) analyzer equipped with special handles, was used for characteristics testing. The head movement speed used was 5 mm·s^−1^. The Flebogrif system was tested for three cutting element protrusion levels: L = L_max_, L = 0.9·L_max_, and L = 0.8·L_max_. Before testing, geometric measurement of the spacing of the cutting elements for three proposed protrusions was performed. It was established that decreasing the working length of the cutting elements will increase their rigidity, and, as a result, increase the force exerted on the internal surface of the vein wall. The obtained characteristics will allow for specifying contact force variability ranges and the corresponding diameter ranges of operated veins.

## 1. Introduction

Chronic venous insufficiency (CVI) is one of the most common diseases globally. It is estimated to occur in 40–60% women and 25–45% men [1]. It is characterized by complex and diverse lesion pathology and type, from telangiectasia and venulectasias, through varicose veins, to thrombotic complications and trophic ulcerations. In most cases, venous hypertension is caused by the insufficiency and reflux of one or more great saphenous veins (GSV), resulting in varicose vein development. Due to the development of endovenous techniques for the last 30 years, stripping has been almost completely replaced with percutaneous procedures. Thermal ablations (endovenous laser ablation—EVLA and radiofrequency ablation—RFA) are strongly recommended by international societies in the treatment of patients with GVS insufficiency and are preferred to surgical treatment and sclerotherapy [2,3]. Due to possible side effects of thermal energy on the adjacent tissues, thermal ablations require careful tumescent anesthesia. Although complications occur rarely, the introduction of non-thermal techniques, including mechano-chemical ablations, have provided new perspectives for treating patients with CVI [4]. Mechano-chemical ablations are less effective than thermal ablations, but they do not cause such complications as burns or nerve damage [5]. MOCA (mechano-chemical ablation) is a recently introduced technique that combines chemical damage through sclerosant foam injection with an endothelial spasm performed by a rotating wire or radial cutting hooks, commercially patented as Clarivein (Merit Medical, Utah, South Jordan, UT, USA) and Flebogrif (Balton, Warsaw, Poland), respectively. The endothelial and medial mechanical damage is purported to enhance the penetration of the sclerosant in the vessel wall and the subsequent vasoconstriction, as proven in ex vivo and animal models [6,7]. Thus, it is reasonable to assume that recanalization rates would be lower than with mechanical or chemical ablation treatments alone. Clarivein has been compared to other techniques such as EVLA and RFA with satisfying results, proving a high safety profile and low recanalization rates [8,9]. Results from a randomized controlled trial published in December 2019 confirm a shorter operative time, lower rates of postoperative phlebitis, and a significantly shorter time to return to work following treatment [10]. The Flebogrif system is a new mechano-chemical ablation method. It consists of five retractable elements with sharp tips for the mechanical damage of endothelium, allowing for the stronger contraction of a vessel and better penetration of a sclerosant into its wall. The spacing of cutting elements of up to 28 mm in diameter, after removing them from the sheath, allows for performing an effective procedure inside veins with a diameter of more than 15 mm, which distinguishes Flebogrif from other thermal and non-thermal ablation techniques. Due to its simple design and low procedure-related costs, it is an interesting option, even more so when considering the good results obtained during a three-year follow-up [11]. Different treatment efficacy depending on the vein diameter prompted the authors to perform tests to establish the maximum system efficacy range and to search for solutions to potentially improve it. Unlike Clarivein, no study has yet been conducted that observes the histological effects of Flebogrif cutting hooks on the endothelium and whether it indeed increases sclerosant penetration in the vessel wall, nor has a comparison been made on its benefit over other comparable techniques.

The purpose of this article was to provide mechanical characteristics in the form of force–displacement dependence for a single cutting element and a bundle of cutting elements of Flebogrif as a whole for different levels of protrusion of the bundle of cutting elements. The results obtained were used to specify the contact force on the vein wall in its diameter function and the protrusion level of the bunch of the cutting elements.

## 2. Materials and Methods

**Figure 1:**
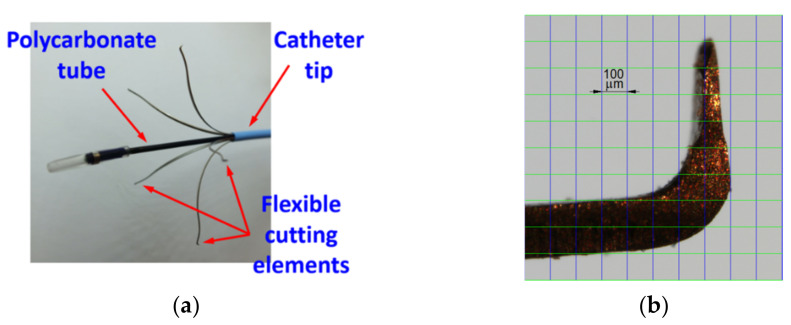
Flebogrif view: (**a**) Tip with five cutting elements; (**b**) Zoom-in of the cutting element.

Four Flebogrif systems (Balton Sp. z o.o., Warsaw, Poland) were tested. Flebogrif is a system for the mechano-chemical ablation of superficial veins in the form of a 90 cm catheter with an internal canal with a polycarbonate tube inside, for which there is a steel clamping ring with five flexible cutting elements at the end. Cutting elements are pre-formed springs that dilate after being removed from the sheath, creating a pattern shown in Figure 1a.

The cutting function is performed by the tips of the flexible elements bundled into a knife shape (Figure 1b), which are located on the vertices of a regular pentagon after expansion. After the arms of the working portion are released, the system is withdrawn in a uniform motion from the positioning site to the vein puncture site while administering 1 mL of 3% polidocanol foam per 5 cm of the treated vein. Scarification depth depends on the pre-shaping and elasticity of the cutting elements, the extent of their protrusion from the sheath, vein diameter, size and shape of the cutting element tips, as well as their angle relative to the inner vein surface. If a cutting element exerts too much pressure on the vein wall, it may cause perforation, while too little pressure may result in a lack of procedure efficacy or premature vessel recanalization. The procedural technique using Flebogrif is described in detail by Ciostek and Zubilewicz [12,13]. Studies by Rybak et al. on an animal model have confirmed the superior efficacy of the system with mechanical ablation supplemented by the effects of a sclerosant [14].

**Figure 2:**
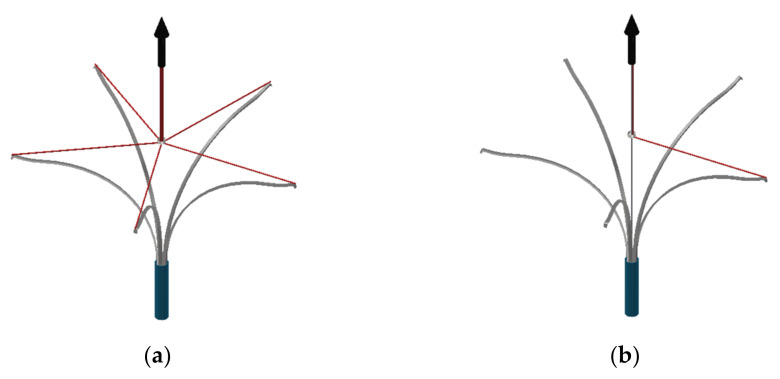
Schematics of the measurement of forces exerted by the Flebogrif cutting elements: (**a**) Measurement of the total force of all cutting elements; (**b**) Measurement of the force of a single cutting element.

To determine the compression force characteristics of the cutting elements, two measurement methods were used, whose concepts are shown in Figure 2. The ends of sections of silk cord were fixed to the cutting element tips and then threaded through a stationary ring. The other ends of the cords were secured in the jaws of a universal testing machine head. The first measurement consisted of the measurement of the total tension of all cords (Figure 2a), and the second consisted of the measurement of the tension of a single cord (Figure 2b). If friction is not considered, the measured force values are the total force acting on all cutting elements (first measurement) and the force acting on a single cutting element (second measurement). However, it should be noted that there is some ambiguity as to the length of the flexible elements, as these lean loosely against the edge of a polycarbonate tube located inside the catheter.

**Figure 3:**
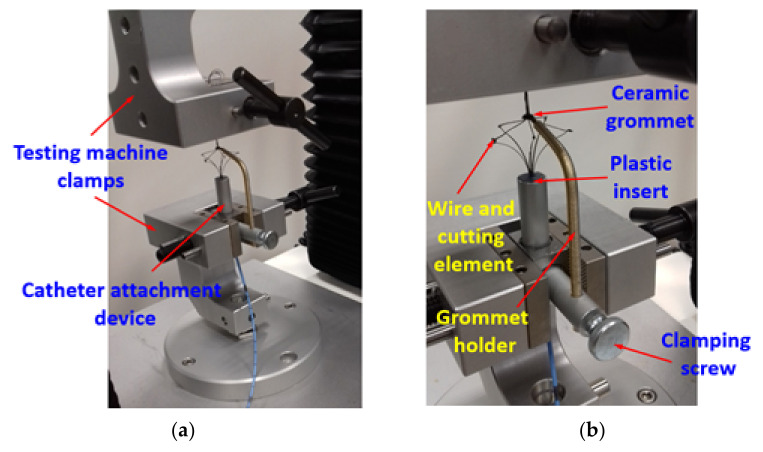
Instrument used to fix the Flebogrif in the measurement position: (**a**) Fixation of the testing instrument in the universal testing machine jaws; (**b**) Structure of the instrument used to fix the Flebogrif tip.

To carry out the measurement item shown in Figure 2, an instrument was used to fix the Flebogrif tip, which is shown together with the Flebogrif in Figure 3.

The structure of the instrument enabled a permanent fixation of the tip while retaining the ability to slide out the central portion of the catheter equipped with steel cutting elements. A plastic insert with a hole was placed in the vertical portion of the instrument that consisted of an aluminum tube. The Flebogrif tip was located inside the insert with the cutting elements protruding. Their tips were bonded using cyanoacrylate glue to sections of silk cord with a diameter of 0.18 mm and a tensile strength of 50 N. The other ends of the tensioning cords were bundled, threaded through a stationary ceramic grommet, and secured in the jaws of a universal testing machine head. Since the length of the system’s cutting elements was changed during testing, the vertical position of the grommet was also adjusted to maintain the horizontal position of the tensioning cords. The grommet position could be changed by vertically sliding the grommet grip, which was fixed to the instrument using a push screw.

**Figure 4:**
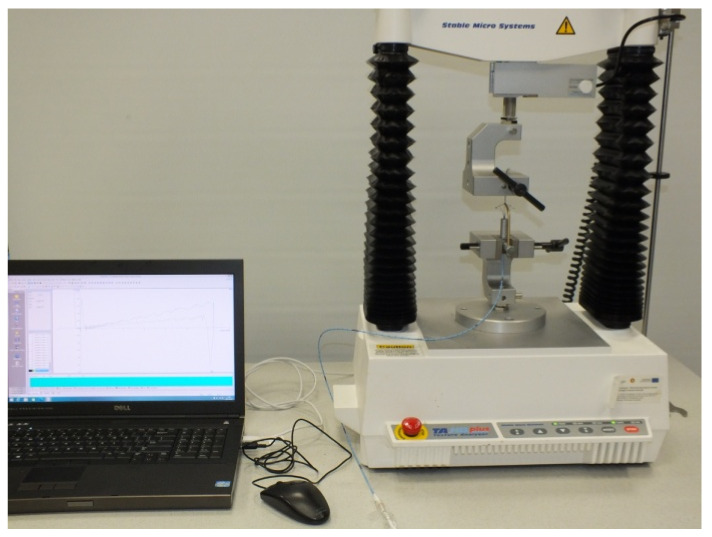
View of the measurement stand.

A TA.HD plus analyzer (Stable Micro Systems, Godalming, UK) was utilized for strength testing (Figure 4). During the experiment, the lower jaw of the universal testing machine remained stationary, while the upper jaw moved at a constant speed of 5 mm⋅s^−1^. Testing was performed for three cutting element protrusion lengths: L = L_max_, L = 0.9⋅L_max_, and L = 0.8⋅L_max_. The protrusion lengths were set at the other end of the catheter by adjusting the shaft protrusion level using the appropriate distance plates. The measurement results were the cord tensile strength characteristics as a function of vertical displacement x of the universal testing machine head.

Strength testing was preceded by measurements of Flebogrif’s geometry. Mean lengths of the diagonals p and sides a of a pentagon (distances between the blades) were determined for the three shaft protrusion levels, L = L_max_ = 18.4 mm, L = 0.9⋅L_max_ = 16.6 mm, and L = 0.8⋅L_max_ = 14.7 mm. Based on the measurement results, the diameter d_f_ of the circumcircle of a regular pentagon was calculated. The values of the pentagon’s diagonals p were substituted in the following relationship:

(1) $$d_{f}=p\cdot \sqrt{50+10\sqrt{5}}\cdot (\sqrt{5}-1)/10,$$

If the measurements of the pentagon’s sides a are used, the diameter of the circumcircle can be calculated using the following formula:

(2) $$d_{f}=a\cdot \sqrt{50+10\sqrt{5}}/5,$$

The results of the measurements and calculations are provided in Table 1.

## 3. Results and Discussion

**Figure 5:**
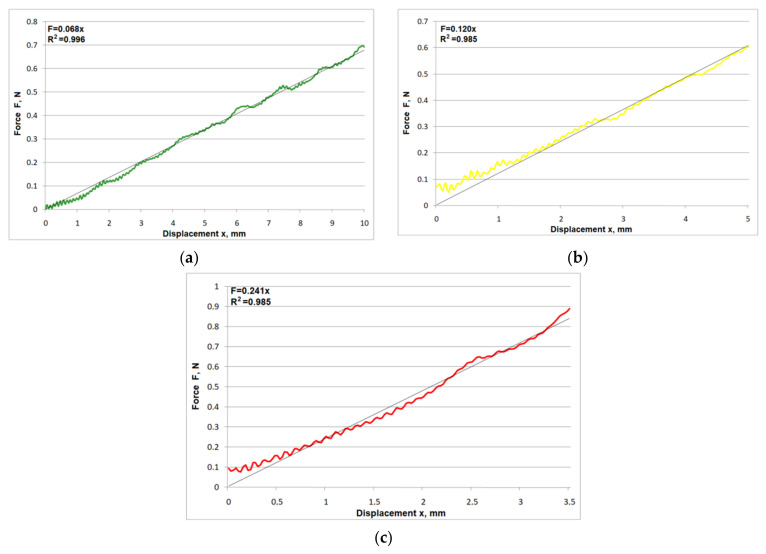
Sample F(x) characteristics for all cutting elements for the following protrusions: (**a**) L = L_max_, (**b**) L = 0.9⋅L_max_, (**c**) L = 0.8⋅L_max_.

Figure 5 shows the typical characteristics of the relationship of force as a function of displacement F(x) for the measurement of total force (acting on all cutting elements) for the different levels of protrusion.

The relationships shown are linear with regard to their values and are characterized by a high correlation coefficient. For the protrusion of L = L_max_, the mean correlation coefficient is R^2^ = 0.995, while for the protrusions of L = 0.9⋅L_max_ and L = 0.8⋅L_max_ the mean R^2^ values were 0.971 and 0.978, respectively. Mean slope values of the straight lines for the protrusion levels used are 0.067, 0.127, and 0.232, respectively. Small rapid fluctuations in the force value are visible throughout the head displacement range characteristics, with an amplitude that does not exceed 0.02 N. These fluctuations are caused by friction between the cord and the ring, and result entirely from the measurement method adopted. These fluctuations do not affect the shape of the characteristics with regard to the mean force values.

**Figure 6:**
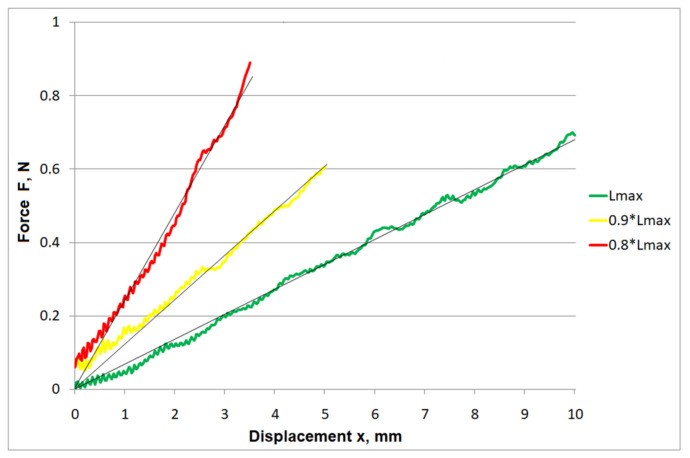
The aggregated F(x) chart for all cutting elements.

A comparison of the force–displacement characteristics obtained for the different lengths of protrusion of the cutting element bundle is shown in Figure 6. The regression lines used to approximate the force distributions show a tendency towards an increase in the slope. This seems wholly justified, as shortening an elastic beam increases its rigidity.

**Figure 7:**
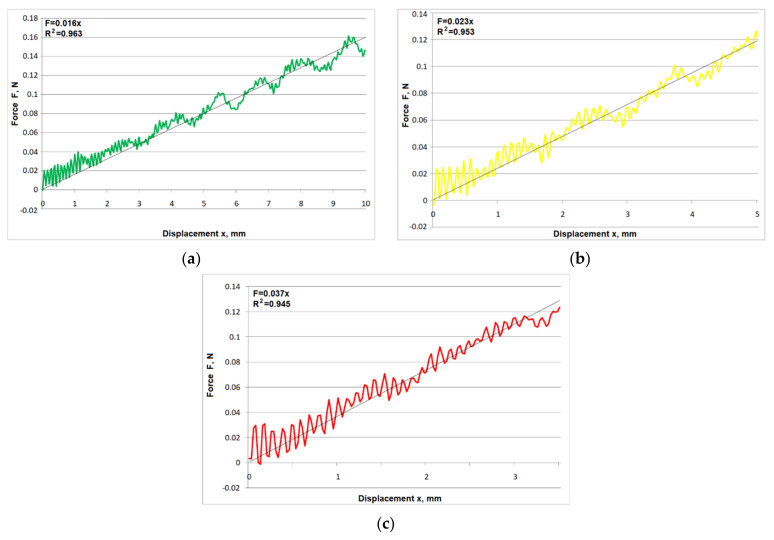
Examples of F(x) characteristics for a single cutting element with protrusion of: (**a**) L = L_max_, (**b**) L = 0.9⋅L_max_, (**c**) L = 0.8⋅L_max_.

Examples of force–displacement characteristics for a single cutting element for different levels of protrusion are presented in Figure 7.

As expected, force–displacement characteristics for single cutting elements are also similar to straight lines. Mean regression coefficient values for subsequent protrusions, starting from the highest, were 0.937, 0.916, and 0.910, respectively. Mean slope values of the straight lines were 0.016, 0.027, and 0.039.

**Figure 8:**
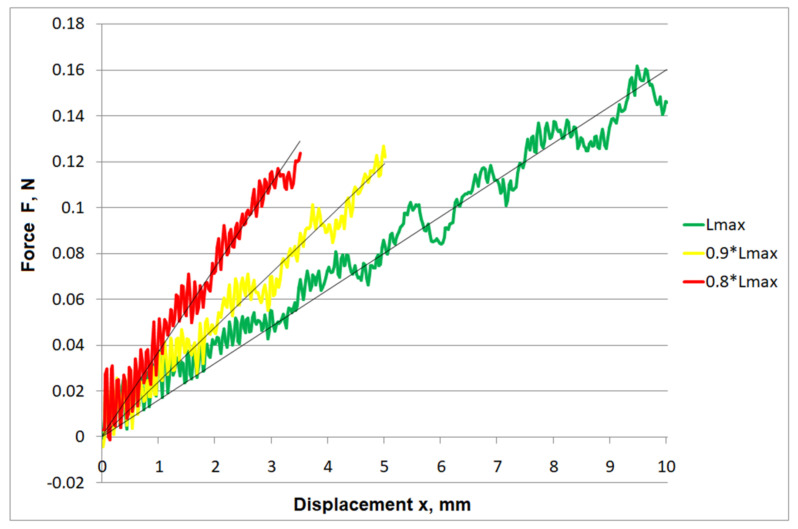
Aggregated F(x) chart for single cutting elements.

The maximum force values obtained for a single cutting element are approximately five-fold lower than the values obtained for all cutting elements (from 4.25 for the maximum protrusion to 5.95 for the protrusion of L = 0.8⋅L_max_). The comparison of force–displacement characteristics obtained for the examples of single cutting elements and the applied protrusions is presented in Figure 8. Similarly, as in the case of the bundle protrusion, the regression lines used to approximate the force distribution for a single cutting element show an upward tendency for decreasing protrusion values.

**Figure 9:**
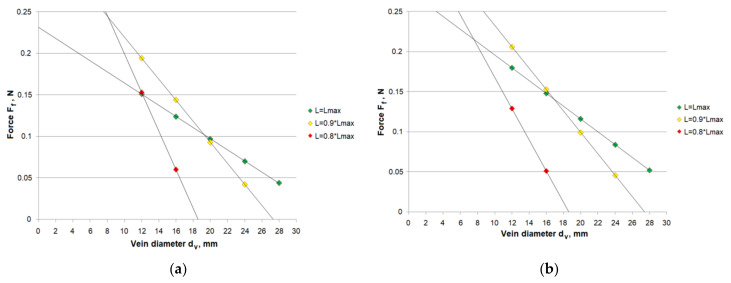
The aggregated F_f_ (d_v_) dependence charts created with the use of: (**a**) the results of force measurements for all cutting elements; (**b**) the results of force measurements for a single cutting element.

Figure 9 shows aggregated charts with characteristics of a single cutting element force acting on the vein wall as a function of the vein diameter for individual Flebogrif protrusions obtained with the use of the measurement results for the total force acting on all cutting elements (Figure 9a), and the forces acting on a single cutting element (Figure 9b). As for the use of measurement results obtained for the total force acting on all cutting elements, the force of a single cutting element acting on a vein wall was calculated using the following formula

(3) $$F_{f}=F/n$$

where F—total force acting on all cutting elements, n—number of cutting elements in a bundle.

In the case of using the results of measurements of the force of a single cutting element, the force acting on the vein wall F_f_ = F. In order to calculate the displacement x, for which a value of the force acting on a vein wall was read, the following relationship was used

(4) $$x=(d_{f}-d_{v})/2$$

where d_f_—the diameter of a circumcircle of the Flebogrif cutting elements, d_v_—vein diameter.

On the basis of the charts presented, it is possible to determine what protrusions should be used to achieve the contact force value falling in the pre-assumed value range. Using the chart in Figure 9a, it may be stated that, e.g., the assumed range of the contact force acting on the vein F_f_ ∈ 〈0.08–0.14〉 N will be achieved for a complete protrusion of the Flebogrif L_max_ and vein diameters between 13.8 and 22.7 mm, the protrusion of 0.9⋅L_max_ and vein diameters between 16.5 and 21.1 mm, and the protrusion of 0.8⋅L_max_ and vein diameters between 12.5 and 15.2 mm.

The introduction of minimally invasive endovenous ablation techniques has revolutionized the GVS insufficiency treatment. Various endovenous techniques have been evaluated in many prospective and randomized studies [8,15,16,17]. Most of them were used for the treatment of the great saphenous vein insufficiency, and only a few for the small saphenous vein (SSV). The results obtained for endothermal techniques proved to be very satisfactory also during the long-term follow-up, with the occlusion rate range being 88–95%. This was shown in the current guidelines, which recommend these methods as the treatment of choice for the GSV insufficiency and, with some limitations, the SSV insufficiency. An alternative solution for thermal ablations was the introduction in Europe in 2010 of the Clarivein system for non-thermal, mechano-chemical ablation that combined mechanical endothelium damage caused by a rotating angled catheter tip with a simultaneous infusion of a liquid sclerosant. Due to the lack of a thermal effect, this method in particular is dedicated to the small saphenous vein treatment [18]. Previous studies show the high efficacy of the method, reaching up to 87–91% during a 3-year follow-up [16,19]. The mechanical injury of the vein wall appears to be the initial and pivotal event that initiates damage of the endothelium and enables penetration of the sclerosant into deeper layers of the venous wall. Mechano-chemical obliteration should be more effective in comparison with standard chemical ablation, since the former should result in a more severe and diffuse inflammatory process that leads to fibrosis of the vein. Ciostek et al. performed a pilot study of the Flebogrif catheter in clinical settings in the years 2011–2013. He included 40 patients presenting with class C2–C6 of chronic venous disease and with incompetence of the great or small saphenous veins demonstrated by a duplex Doppler. Thirty-nine patients completed the study. The efficacy of the procedure—defined as occlusion of the treated saphenous vein—assessed at follow-up visits was: after one month 97.4% (38/39), after 3 months 94.9% (37/39), after 6 months 89.7% (35/39), and after 12 months 89.7% (35/39) [12].

In two other studies, the Flebogrif system presents similar to the Clarivein 3-year efficacy of 92% and clinical success demonstrated by improvement of VCSS (Venous Clinical Severity Score) [11,20]. Only one study compared Flebogrif with EVLA in a randomized controlled trial, with occlusion rates of 96% vs. 98%, respectively [10]. The development of deep vein thrombosis after Flebogrif occurred in 0.3% of the patients within 12 months of follow-up. The data on ClariVein reported deep vein thrombosis in 0.0% to 1.0% of patients [21].

Due to the number of recanalizations increasing with the increase in the treated vein diameter, the maximum transverse diameter was considered to be 15 mm [22]. Naturally the treatment of wider veins is also possible; however, it usually requires the use of higher energy, very carefully performed tumescence, and is also associated with a higher risk of recurrence.

The performed experiment showed that the maximum effect was achieved for all tested ranges of the cutting element spacing with vein diameters of 12.5–22.7 mm.

Due to the wide cutting element spacing, and the comparable and strong mechanical effect on the vein wall shown in the study, the Flebogrif system can be effectively used in a much broader vein diameter range when compared to alternative endovenous treatment methods [23].

## 4. Conclusions

1. The method established and used to measure the acting force both for the cutting element bundle and for a single cutting element allowed for an effective determination of their mechanical characteristics.
2. The tests allowed for the determination of the value range for the Flebogrif cutting element contact force acting on the vein surface, depending on the protrusion of the system from the sheath introducer.
3. Decreasing the working length of the Flebogrif cutting elements results in an increase in their rigidity, which is associated with the increase in contact forces acting on the internal surface of the vein. As a result, this leads to the determination of the force variability ranges and corresponding diameter ranges of operated veins.
4. On the basis of Flebogrif testing, it may be stated that the values of contact force acting on a vein falling in the range of F_f_ ∈ 〈0.08–0.14〉 N can be achieved for a complete catheter protrusion of L_max_ and vein diameters between 13.8 and 22.7 mm, the protrusion of 0.9⋅L_max_ and vein diameters between 16.5 and 21.1 mm, and the protrusion of 0.8⋅L_max_ and vein diameters between 12.5 and 15.2 mm.

## Figures and Tables

**Table 1:** Results of measurements and calculations of Flebogrif geometrical value.

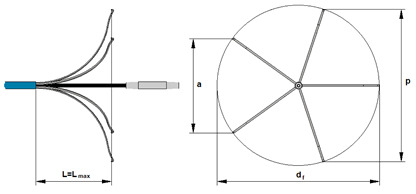			
Protrusion Levels L	Side a (mm)	Diagonal p (mm)	Calculated Diameter d_f_ (mm)
L = L_max_	20.6	33.2	34.5
	21.5	32.5	
	19.8	31.8	
	20.5	32.9	
	19.6	32.5	
L = 0.9⋅L_max_	16.3	25.2	27.3
	17.2	25.1	
	16.5	24.8	
	17.1	25.2	
	16.4	24.6	
L = 0.8⋅L_max_	11.0	17.7	18.6
	11.3	17.8	
	10.8	17.6	
	11.2	17.7	
	10.1	17.7	

## Data Availability

Data are contained within the article.
